# USH1G with unique retinal findings caused by a novel truncating mutation identified by genome-wide linkage analysis

**Published:** 2012-07-12

**Authors:** Faiqa Imtiaz, Khalid Taibah, Ghada Bin-Khamis, Shelley Kennedy, Amal Hemidan, Faisal Al-Qahtani, Khalid Tabbara, Bashayer Al Mubarak, Khushnooda Ramzan, Brian F. Meyer, Mohammed Al-Owain

**Affiliations:** 1 Department of Genetics, King Faisal Specialist Hospital & Research Centre, Riyadh, Saudi Arabia; 2 ENT Medical Centre, Riyadh, Saudi Arabia; 3 Department of Otolaryngology, King Faisal Specialist Hospital & Research Centre, Riyadh, Saudi Arabia; 4 Ontario Newborn Screening Program, Children's Hospital of Eastern Ontario, Ottawa, Ontario, Canada; 5 Department of Ophthalmology, King Faisal Specialist Hospital & Research Centre, Riyadh, Saudi Arabia; 6 Department of Medical Genetics, King Faisal Specialist Hospital & Research Centre, Riyadh, Saudi Arabia; 7 College of Medicine, Al-Faisal University, Riyadh, Saudi Arabia

## Abstract

**Purpose:**

Usher syndrome (USH) is an autosomal recessive disorder divided into three distinct clinical subtypes based on the severity of the hearing loss, manifestation of vestibular dysfunction, and the age of onset of retinitis pigmentosa and visual symptoms. To date, mutations in seven different genes have been reported to cause USH type 1 (USH1), the most severe form. Patients diagnosed with USH1 are known to be ideal candidates to benefit from cochlear implantation.

**Methods:**

Genome-wide linkage analysis using Affymetrix GeneChip Human Mapping 10K arrays were performed in three cochlear implanted Saudi siblings born from a consanguineous marriage, clinically diagnosed with USH1 by comprehensive clinical, audiological, and ophthalmological examinations. From the linkage results, the *USH1G* gene was screened for mutations by direct sequencing of the coding exons.

**Results:**

We report the identification of a novel p.S243X truncating mutation in *USH1G* that segregated with the disease phenotype and was not present in 300 ethnically matched normal controls. We also report on the novel retinal findings and the outcome of cochlear implantation in the affected individuals.

**Conclusions:**

In addition to reporting a novel truncating mutation, this report expands the retinal phenotype in USH1G and presents the first report of successful cochlear implants in this disease.

## Introduction

Usher syndrome (USH) is an autosomal recessive disorder that is clinically and genetically heterogeneous associated with sensorineural hearing impairment and progressive visual loss attributable to retinitis pigmentosa (RP). USH is the most common cause of hereditary deaf-blindness, reported in 1 in 6,000 children [1].

Usher type 1 (USH1) is the most severe of three known clinical subtypes as patients affected have severe to profound congenital hearing loss combined with prepubertal onset of visual symptoms. In addition, individuals with USH1 often walk later than usual due to vestibular dysfunction, and older children with USH1 may appear clumsy and have difficulty with gross motor activities that require a level of balance. To date, seven loci have been mapped that cause USH1, USH1B–USH1H (Hereditary Hearing Loss Homepage).

The locus for USH1G was mapped to 17q24–25 [2], and in 2003 the gene *USH1G* (previously named scaffold protein containing ankyrin repeats and sam domain [*SANS*]) was cloned, which is the human ortholog of the *Sans* gene defective in Jackson shaker mutant mice [3,4]. *USH1G* contains three exons, spans 7.2 kb, and encodes a scaffolding protein (SANS) with 460 amino acids. The SANS protein contains three ankyrin domains at the N-terminal end (amino acids 31–129) and a PDZ-binding motif at the C-terminal end. In-between lies a central region (amino acids 130–385) and a sterile alpha motif (SAM) domain (amino acids 384–446) [3]. Since then, only a small number of patients with *USH1G* mutations have been reported in the literature with prelingual hearing loss, vestibular dysfunction, and variable RP [3,5-7].

In this report, we describe three siblings from a consanguineous family of Saudi Arabian origin with USH1G and distinct retinopathy, who also had a good outcome after cochlear implantation.

## Methods

### Patient information and clinical evaluation

**Figure 1:**
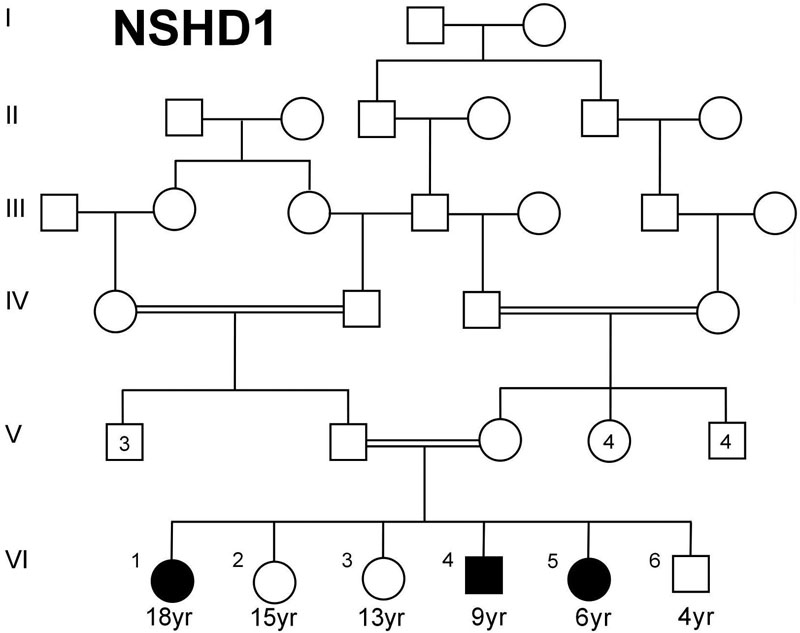
The pedigree of the Usher family with three affected siblings and an autosomal recessive pattern of inheritance.

All of the individuals who participated in this study provided an approved informed consent form, which adhered to institutional (King Faisal Specialist Hospital; RAC# 2040039) guidelines and to the tenets of the Declaration of Helsinki. Three siblings affected with hearing loss and RP from a consanguineous family (Figure 1) of Saudi Arabian origin were recruited for this study. Detailed clinical and developmental histories were obtained for all the members of this family.

Hearing was assessed both pre- and post-cochlear implantation for all three patients by pure tone visual reinforcement audiometry; air conduction and bone conduction thresholds were measured at frequencies 250, 500, 1,000, 2,000, 4,000, and 8,000 Hz in a sound booth with a Grason-Stadler Diagnostic Clinical Audiometer (Grason-Stadler, Eden Prairie, MN). In addition, diagnostic brainstem evoked response audiometry was performed using click stimulation. Dilated funduscopy and electroretinography (ERG) and visual field were performed for ophthalmological examinations. Vestibular function was evaluated by testing tandem gait ability and by using the Romberg test.

### Sample collection and DNA extraction

Whole venous blood samples (10 ml) were collected and immediately processed for genomic DNA extraction from peripheral blood leucocytes, using the standard protocols. These were obtained from the three patients described above, their parents, and two unaffected siblings. Genomic extraction of DNA was performed using the standard salting-out method [8].

### Linkage analysis

SNP-based genotyping was performed using the Affymetrix GeneChip Human Mapping 10K arrays (Affymetrix, Santa Clara, CA). The genotypes of single nucleotide polymorphisms (SNPs) were called using Affymetrix GCOS 1.4 software, which generated an overall average SNP call rate of 97%. The Allegro module of the Easy Linkage software package was used to calculate multipoint logarithm of the odds (LOD) scores, with the parameters that assume a disease model with an autosomal-recessive mode of inheritance with 100% penetrance and a disease allele frequency of 0.0001.

### Mutation screening in *USH1G*

Genomic DNA of all individuals was amplified by PCR using intronic primers that were designed to flank each of the three coding exons of *USH1G* (Table 1). PCR was performed in a final volume of 20 µl containing approximately 20 ng of genomic DNA, 50 mM KCl, 10 mM Tris-HCl (pH 8.3), 1.5 mM MgCl_2_, 200 µM deoxyribonucleotide triphosphates (dNTPs), 1 unit of Qiagen (Valencia, CA) HotStar Taq polymerase, and 10 µM of each primer. Thermocycling (Applied Biosystems Inc., Foster City, CA) consisted of an initial denaturation at 95 °C for 15 min followed by 35 cycles of PCR. Each cycle of PCR consisted of denaturation at 94 °C for 60 s, annealing at 62 °C for 60 s and extension at 72 °C for 60 s. A final extension step of 10 min at 72 °C was added.

**Table 1:** PCR primers for the three coding exons of the *USH1G* gene.

**Primers**	**Forward**	**Reverse**
Exon 1	CATGCCTCAGCCCTAATACC	AGCTCAGAGGAGTGGTGGAC
Exon 2a	TGCTGTGACAGTGGGGAAG	CGTGGCCTGAGAGTACGG
Exon 2b	ACACCCTCAGCTTCTCCAG	AGGCTGTCATCGTCCAGG
Exon 2c	ACGACTCCCTGTTTACCCG	CCTGAATAGGCAGATCTGTACC
Exon 3	ATGGGGAGGCTAAGTTGTCC	CAACTGTGAGGACCTCGAGAC

### Automated sequencing

Purified PCR products covering the entire coding region of *USH1G* as identified on the UCSC and Ensembl websites, were directly sequenced with the dideoxy chain-termination method using an ABI Prism Big Dye Terminator v3.1 Cycle Sequencing Kit following the manufacturer’s instructions, and processed on a MegaBACE 1000 DNA Analysis System (Molecular Dynamics; Sunnyvale, CA). Sequence analysis was performed using the SeqMan 6.1 module of the Lasergene (DNA Star Inc.; Madison, WI) software package, and then compared to the reference sequence (GenBank NG_007882). Numbering commenced with the A of the ATG initiation codon as +1.

## Results

### Clinical description

At the time of study, the father of the proband was 41 years old, and the mother was 36 years old. Both were reported to be in good health. The parents were first cousins, related through their fathers who were half-brothers. They had six children in total, three of whom had hearing loss. There was no family history of recognizable genetic conditions, birth defects, or mental retardation. A comprehensive review of the extended family pedigree did not reveal any other individuals with hearing loss.

#### Patient 1

The proband was 18 years of age at the time of study. She was born after an uncomplicated pregnancy. Her hearing loss was confirmed at 10 months of age via auditory brainstem evoked response testing. She was initially fitted with hearing aids and subsequently had a cochlear implant at 6 years of age. She had delayed motor milestones (she sat and walked at 1 year and 2 1/2 years of age, respectively) and expressive speech delay, but she was cognitively normal. She had a positive Romberg test and can walk in tandem gait. She had problems in visual fixation, such as in reading during walking and feeling insecure when walking in unfamiliar areas like walking on a sandy beach. Night-blindness was noted as the first indication of retinal degeneration. She is currently earning her bachelor’s degree in business administration.

#### Patient 2

At the time of enrollment, the younger brother of the proband was 9 years old. He was delivered after an uncomplicated pregnancy by Cesarean section due to fetal distress. His birthweight was 2.75 kg. His hearing loss was detected shortly after birth, and he was fitted with hearing aids at 9 months of age. A computed tomography scan of his temporal bones was normal, as was his toxoplasmosis, rubella, cytomegalovirus, herpes simplex, and HIV (TORCH) screen. He subsequently had a cochlear implant at 2 years of age. An ophthalmology examination at 14 months was normal with no evidence of retinopathy. His early motor milestones were delayed (sitting and walking at 1 and 3 1/2 years of age, respectively); however, a developmental assessment at 3 1/2 years showed normal cognition with moderate speech delay. The most severely affected of three affected siblings, he was a late walker and has a clumsy ataxic gait with frequent falls, especially in unfamiliar areas. As a result, his family was afraid that he might cause injury to himself. He had a positive Romberg test, and cannot perform tandem gait. Patient 2 is currently in the fourth grade in a normal school and is doing well.

#### Patient 3

The proband’s sister was reported to have the mildest phenotype and was 6 years of age at the time of enrollment. She was born vaginally after an uncomplicated pregnancy. Her hearing loss was detected 2 weeks after birth by brainstem auditory evoked potential. She had a cochlear implant at 2 years of age. She sat at 9 months and walked at 18 months of age. At 2 1/2 years, her developmental assessment noted she was clumsy and prone to falling. Her cognitive development was normal, but she had severe speech and language delay. This patient is currently in elementary school and is reported to be doing well.

### Audiological evaluation and cochlear implantation

**Figure 2:**
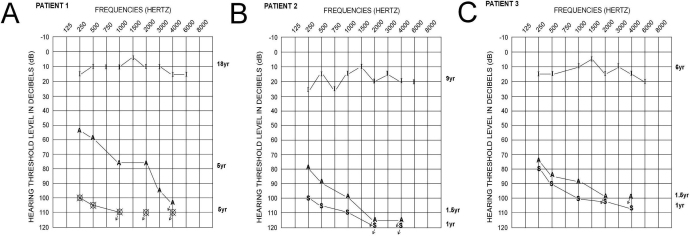
Audiometry. Audiological evaluation of Patient 1 (**A**), Patient 2 (**B**), and Patient 3 (**C**) shows the hearing thresholds at all tested frequencies pre- and post-cochlear implantation. Pure tone sound field audiograms are represented by unaided (S), Aided (A), and Implanted (I). “O” and “X” symbols correspond to the right and left ear pure tone air conduction audiograms, respectively. Audiometric measurements were obtained at specific ages for each patient as denoted by each hearing assessment. All three affected individuals had bilateral profound sensorineural hearing loss before cochlear implantation. Post-implantation, all affected acquired hearing threshold measurements in the normal range.

All three affected individuals were diagnosed with bilateral congenital prelingual profound sensorineural hearing loss and consequently received cochlear implants (Figure 2).

The proband received a Cochlear Nucleus 22 cochlear implant at the age of 6 years; her final aural rehabilitation session was in November, 2006 when she was able to comprehend open-sent speech, answer Wh-questions from an open set, participate effectively in group conversation, and discriminate on the telephone with a familiar person. Comprehending and producing Wh-questions are crucial abilities in communication, for example, Wh-questions that begin with *which* and *who* and the ability of orally trained deaf children to understand and produce other structures that involve the same syntactic construction, Wh-movement [9]. Patient 2 had a Nucleus 24 Contour cochlear implant at the age o 23 months. At the age of 9 years, the child had met the long-term goals of the aural rehabilitation program as he was able to comprehend from an open set, answer Wh-questions from an open set, participate effectively in group conversation, as well as to discriminate on the telephone with unfamiliar persons, although he still had some difficulties with gender markers (feminine versus masculine). Patient 3 received a Nucleus 24 Contour cochlear implant at age 16 months. At the age of 6 years, the child had met the long-term goals of the program as she was able to comprehend from an open set, answers Wh-questions from an open set, participate effectively in group conversation, and was able to discriminate on the telephone with unfamiliar persons.

### Retinal description for the *USH1G* family

**Figure 3:**
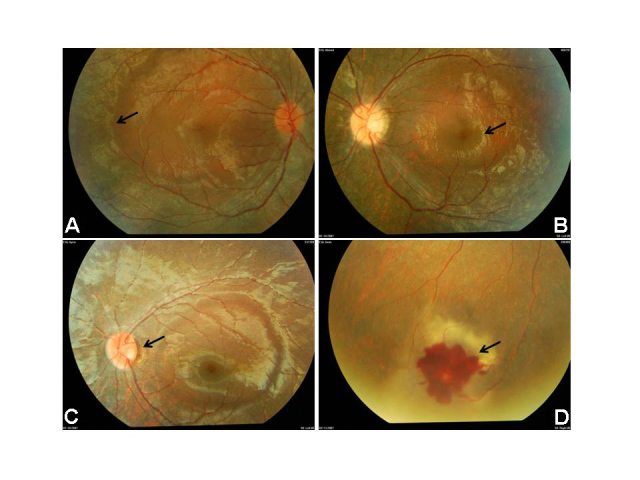
Novel retinal findings. Panels **A** and **B** are of fundus photos showing a normal-looking macula and an abnormally mottled retinal pigment epithelium in the periphery. The photo in Panel **C** shows the demarcation line circular, separating the normal-looking macula from abnormal periphery, mild optic nerve atrophy; optic nerve pit and attenuation of retinal arterioles could be seen (Patient 3). Panel **D** shows the proband’s local macroaneurysm with limited retinal hemorrhage.

**Figure 4:**
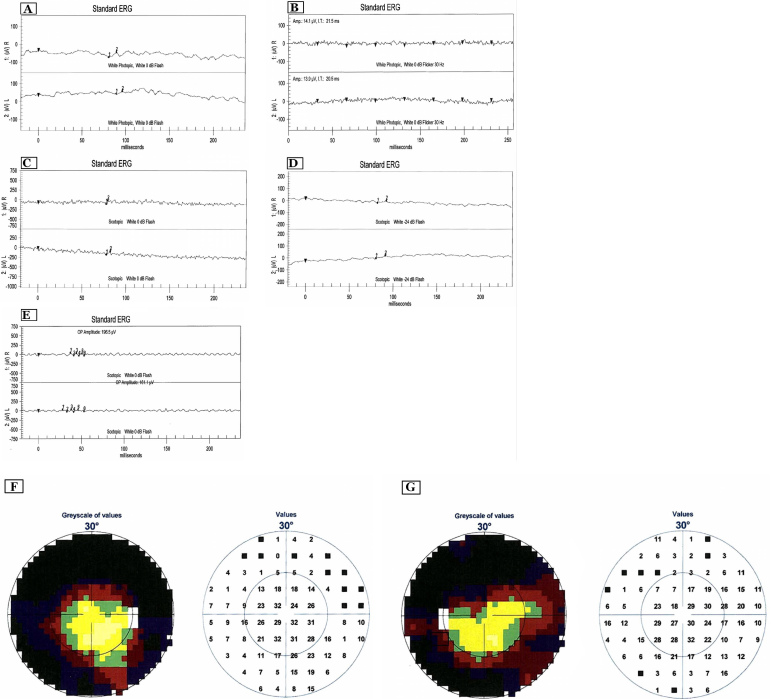
Figures depicting the results of ERG and visual field testing in the proband. **A**: Results of ERG testing showed severe attenuation of phobic (**A** and **B**) and scotopic (**C**, **D**, **E**) waveforms in the proband. **F, G**: Visual field testing. Visual field testing using the Octopus screening program showing severe generalized restriction of the peripheral field in the proband.

All three siblings had a normal-looking macula surrounded by an abnormal peripheral atrophic retina that had an abnormal light reflex with a mottled retinal pigment epithelium without any pigment migration. There was a well demarcated line in a circular fashion separating the normal-looking macula and the abnormal periphery (Figure 3C). Patient 2 showed more severe changes in the form of a smaller circle of a normal-looking macula, mild optic atrophy, and attenuated arterioles (Figure 3C). The youngest sibling (Patient 3) had similar severe retinal changes with a left temporal optic nerve pit and mild optic pallor (Figure 3C). The proband had peripheral isolated retinal vascular telangiectasia with local ischemia surrounding a macroaneurysm that had caused a limited retinal hemorrhage (Figure 3D). Her fluorescein angiogram showed delayed choroidal filling underneath the abnormal retina with normal choroidal filling in the macular area. There was a “starry sky” appearance in the periphery. The macroaneurysm was demonstrated by early point hyperfluorescence through the retinal hemorrhage with a feeder artery and late leakage. All three siblings had normal visual acuity but constricted visual fields (Figures 4A,B) as shown by the visual field testing. The ERG (LKC Technologies, Inc., Gaithersburg, MD) was flat in all three affected individuals. Although the macula looked normal, the cone function was not seen on a standard ERG. A focal ERG was not available, and it might have shown normal macular function.

### Disease locus identification

**Figure 5:**
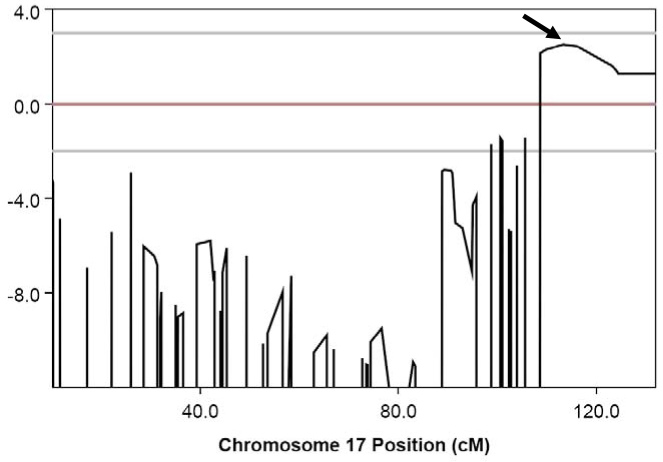
Results of linkage analysis in the Usher family showing a LOD score of 2.5 on chromosome 17. Linkage analysis reveals a LOD score of 2.5 on chromosome 17q26.3, as indicated by an arrow.

The resulting multipoint linkage analysis of the three affected patients, their three unaffected siblings, and their parents identified a disease locus on chromosome 17q24.3-q25.3 (Ensembl cytogenetic band) with a maximum logarithm of odds of 2.5 (Figure 5) between SNP markers rs718072 and rs2333990, which spanned an 8.3 Mb linkage interval. The *USH1G* gene (NM_173477) was selected as the first choice from within this interval as the most likely disease-causing candidate.

### Mutation detection in *USH1G*

**Figure 6:**
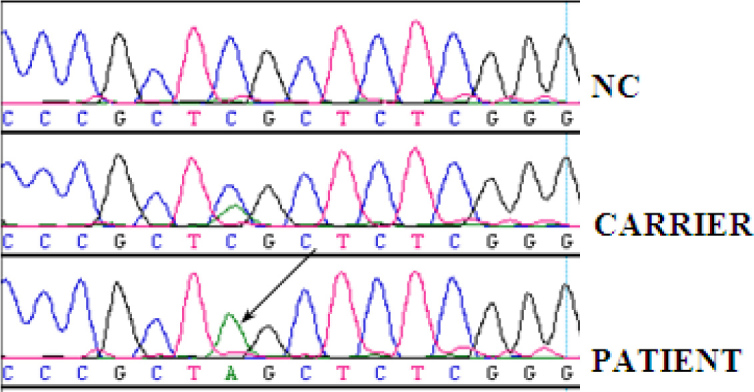
Mutation analysis-sequence data Sequence chromatogram of a normal control (NC), an individual heterozygous for the c.728C >A (p.S243X) mutation (CARRIER) and a homozygous p.S243X (indicated by an arrow) mutation in an affected patient (PATIENT).

Direct sequencing in the forward and reverse directions identified a homozygous truncating mutation, c.728C > A (p.S243X), in all three USH1 affected siblings (Figure 6). The mutation segregated with the disease phenotype (both parents and all three unaffected siblings were heterozygous carriers). The p.S243X mutation was not found in 300 ethnically matched normal controls, which signified that this variant was not a population-based polymorphism.

The URLs for the websites used in this manuscript are the UCSC Genome Browser, Hereditary Hearing Loss Homepage, and Ensembl Genome Browser.

## Discussion

USH1G is a rare cause of USH1 with an estimated frequency of about 7% [5]. Roux and colleagues [10] screened 34 families with deafness and identified no mutation in *USH1G*. To the best of our knowledge, we describe the first Saudi family with USH1G using SNP-based linkage technology. Clearly, the cohort has the typical findings of USH1 with the hearing loss, retinopathy, and vestibular dysfunction. The genome-wide linkage analysis identified a region on chromosome 17q26.3 that contained the known USH1-causing gene, *USH1G*. Direct sequencing analysis of *USH1G* detected the presence of a novel p.S243X truncating mutation that segregated with the disease phenotype in the family and was not found in 300 ethnically matched normal controls. All three siblings have received successful cochlear implants and are living considerably normal lives with good educational performance. This is, to our knowledge, the first report in the literature of successful cochlear implants in patients with USH1G. Based on the Human Gene Disease Mutation Database (HGMD) at the Institute of Medical Genetics in Cardiff, UK, seven mutations (Table 2) have been reported (three missense/nonsense, three small deletions, and one small insertion).

**Table 2:** Comparison of molecular, audiological, ophthalmological and clinical findings in all reported USH1G patients to date.

**Clinical, genetic and patient information**	**Weil et al. [****3****]**	**Ouyang et al. [****5****]**	**Kalay et al. [****6****]**	**Mustapha et al. [****2****]**	**Weil et al. [****3****]**	**Bashir et al. [****7****]**	**Present study**
Mutation	c.142C>T (p.L48P)/186–187delCA	c.113G>A (p.W38X)	c. 1373A>T (p.D458V)	c.832–851del20	c.393insG	c.163_164+13del15	c. 728C>A (p.S243X)
Consequences	Missense/frameshift	Nonsense	Missense	Frameshift	Frameshift	Frameshift	Nonsense
Country	Germany	USA	Turkey	Jordan	Tunisia	Pakistan	Saudi Arabia
Number of cases	2 (familial)	2(sporadic)	6 (familial)	3 (familial)	8 (familial)	4 (familial)	3 (familial)
Hearing loss	Profound	Profound	Prelingual (moderate to profound) HL	Prelingual profound HL	Congenital profound HL	Moderate to severe HL	Congenital profound HL
Visual acuity	ND	ND	Normal	ND	ND	Mild loss of near-sight vision	Normal with very constricted visual fields
Funduscopy	ND	ND	Variable bone spicules and peripheral retinal pigmentary atrophy. No waxy pallor of optic discs, mild RP	Variable RP	Severe RP	Pale optic discs, mild RP	Normally looking macula surrounded by an abnormal peripheral atrophic retina. No evidence of pigmentary migration
ERG	ND	ND	ND	Variably severe retinal degeneration	Severe RP	ND	Flat in all members
Vestibular function	ND	ND	Normal	Abnormal	Abnormal	Normal	Abnormal
Cochlear implant	ND	ND	ND	ND	ND	ND	Successful

ND: Not determined.

There is clearly a variable severity of the retinopathy in the patients with USH1G reported in the literature (Table 2). In fact, the patients reported by Kalay et al. [6] (ages 13, 18, 20, 22 years) had no visual symptoms or night blindness. Moreover, the patients’ visual acuity was normal. However, the ERG of another 4-year-old child with USH1G showed severe retinal degeneration [2]. The retinal findings in the three siblings in this report are a distinct form of RP. The normal-appearing macula with a well defined line/junction separating the macula from the abnormal peripheral retinal reflex points to the atypical retinopathy in this family. Interestingly, the two younger patients had more severe retinopathy than the older proband. The optic nerve pit and the peripheral telangiectasia were isolated findings in two separate patients, which may be an unusual part of the phenotype. Compared to the findings of Kalay et al. [6], there was no pigment migration (bony spicules), which may give a hint that the peripheral cells are more preferentially affected in this family.

SANS has a highly conserved function in vesicle trafficking among species [3]. It is expressed in lens-secreting cone cells of the Drosophila adult eye [11]. In Jackson shaker mice with defective *Sans*, harmonin is completely absent in the hair cells of the inner ear starting early at the embryonic stage [3,4]. This indicates an important role of SANS in the trafficking of harmonin to the stereocilia of the hair cells [12], the essential mechanosensitive devices for detecting sound [12,13]. In cotransfection experiments, *Sans* was also shown to associate with harmonin [14]. Recently, the two proteins were discovered to form a highly stable complex structure, the formation of which is disrupted in patients with USH1 mutations of SANS and harmonin [15]. In the mammalian eye, the interaction between SANS and whirlin (USH2D) in the apical inner segment collar and the ciliary apparatus of the photoreceptor cells was described [16]. In addition, SANS provides a linkage to the microtubule transport machinery [15]. In 2011, using Ush1g knockout mice experiments, Caberlotto and colleagues [17] concluded that Sans belongs to the USH1 protein network required for the cohesion of hair bundles in cochlear hair cells during the early stages of their development. These authors [17] also confirmed that Sans localizes to the lower end of tip-links, which interconnect the stereocilia of the hair bundle and function as controllers of the mechanoelectrical transduction channels in these auditory hair cells [17]. Concurrently in 2011, Grati and Kachar [18-20], using immunofluorescence intensity techniques, demonstrated MYO7A and Sans in fact cluster at the stereocilia upper tip-link density, which is suggested to contain a cluster of various myosin motor proteins that pull on the tip-link to maintain resting tension.

The p.S243X in this family is located in the central region of SANS, which directly interacts with the tail domain of myosin VIIa (MYO7A) [21], thereby linking harmonin with the actin-based motor [14]. In addition, the central region along with the ankyrin repeats play an important role in the cytoplasmic puncta formation of SANS [14]. MYO7A has been shown to be expressed in the photoreceptor cilium and the adjacent retina pigmented epithelium [22-25], and defects in *MYO7A* cause USH1B [26].

The localization of this nonsense mutation may explain why the retinal findings in our patients are different from the D458V mutation affecting the C-terminal tail of SANS [6]. Moreover, the p.S243X and in fact all of the mutations reported to date, apart from D458V, are predicted to result in premature termination codons (PTCs), causing aberrant mRNAs that encode truncated proteins. These PTCs are known to be targets for elimination by a regulatory and specialized surveillance mechanism, known as nonsense-mediated mRNA decay (NMD) [27]. It has previously been estimated that approximately one-third of all genetic diseases are caused by variations causing PTCs and NMD is in fact a crucial factor in modulating inherited human disease phenotypes [28-31]. NMD can be of benefit and perhaps reduce disease severity, for example, in removing aberrant transcripts that encode proteins with dominant-negative effects. However, NMD may be a disadvantage if truncated proteins sustaining some normal function are eliminated [31,32]. Thus, the wide variability in the retinal phenotype in all USH1G patients to date may be partly due to the predicted NMD outcome of each PTC, with respect to its position in the SANS protein.

Previously, nonsense mutations resulting in USH1C [33] and USH1F [34,35] have been the targets of in vitro and in vivo experiments designed specifically with potential therapeutic possibilities using various compounds, including aminoglycosides [33], and derivatives of the clinical aminoglycoside paromomycin [35,36]. These antibiotics have the unique capability to promote translation read-through of PTCs but not of normal termination codons, resulting in restoration of normally functioning proteins [36-39]. Such efforts resulted in restoration of protein function in both of the Usher genes targeted, and it would be of future interest to determine whether the premature truncation of SANS caused by USH1G nonsense mutations reported to date are restored using similar strategies.

Finally, in addition to the molecular, audiological, and ophthalmological characterization of the siblings described in this study, the identification of the novel pathogenic mutation described has subsequently proven to be of tremendous use to this family and the extended family, with respect to inductive carrier testing and premarital screening.
